# Case Report: A Rare Case of Esophagogastric Junctional Squamous Cell Carcinoma After the Successful Treatment of Neuroendocrine Carcinoma: Clonal Tumor Evolution Revealed by Genetic Analysis

**DOI:** 10.3389/fgene.2021.608324

**Published:** 2021-09-15

**Authors:** Hiroki Sato, Takeshi Saito, Hiroshi Horii, Mami Kajiura, Noriaki Kikuchi, Nobuhisa Takada, Koichi Taguchi, Mika Yoshida, Masakazu Hasegawa, Hiroyuki Taguchi, Yukinori Yoshida, Katsuyoshi Ando, Mikihiro Fujiya, Yuko Omori, Thomas Hank, Andrew S. Liss, Manish K. Gala, Yoshio Makita, Yusuke Ono, Yusuke Mizukami, Toshikatsu Okumura

**Affiliations:** ^1^Division of Internal Medicine, Sunagawa City Medical Center, Sunagawa, Japan; ^2^Division of Metabolism and Biosystemic Science, Gastroenterology, and Hematology/Oncology, Department of Medicine, Asahikawa Medical University, Asahikawa, Japan; ^3^Division of General and Gastrointestinal Surgery, Massachusetts General Hospital and Harvard Medical School, Boston, MA, United States; ^4^Division of Pathology, Sunagawa City Medical Center, Sunagawa, Japan; ^5^Department of Investigative Pathology, Tohoku University Graduate School of Medicine, Sendai, Japan; ^6^Gastrointestinal Unit, Massachusetts General Hospital and Harvard Medical School, Boston, MA, United States; ^7^Department of Genetic Counseling, Asahikawa Medical University Hospital, Asahikawa, Japan; ^8^Institute of Biomedical Research, Sapporo Higashi Tokushukai Hospital, Sapporo, Japan

**Keywords:** genetic pathology, multidisciplinary therapy, sequential chemotherapy, neuroendocrine carcinoma, squamous cell carcinoma, tumor genotyping, clinicopathological correlation, esophagogastric junction

## Abstract

Neuroendocrine carcinoma (NEC) of the esophagogastric junction (EGJ) is a rare disease with no established treatments. Herein, we describe a case of recurrent squamous cell carcinoma (SCC) after achieving complete response to chemotherapy against NEC of the EGJ. A 67-year-old man was referred to our hospital because of epigastric discomfort. Computed tomography imaging and esophagogastroduodenoscopy revealed ulcerated tumors at the EGJ. Endoscopic biopsy revealed small tumor cells with a high nuclear/cytoplasmic ratio, suggesting small-cell NEC. Immunohistochemistry (IHC) analysis showed tumor cells with an MIB-1 index of 80%. The patient achieved complete response after 10 cycles of chemotherapy. Follow-up endoscopic examination revealed small red-colored mucosal lesions in the center of the cicatrized primary lesion. Re-biopsy detected cancer cells harboring large eosinophilic cytoplasm with keratinization and no evidence of NEC components. IHC of the cells were cytokeratin 5/6-positive and p53-negative. The tumor persisted without evidence of metastases after chemoradiotherapy, and total gastrectomy with lymph node dissection was performed. Pathological assessment of the resected specimens revealed SCC, without evidence of NEC. The patient survived without a recurrence for >3 years after the initial presentation. Somatic mutation profiles of the primary NEC and recurrent SCC were analyzed by targeted amplicon sequencing covering common cancer-related mutations. Both tumors possessed *TP53* Q192X mutation, whereas *SMAD4* S517T was found only in SCC, suggesting that both tumor components originated from a founder clone with a stop-gain mutation in *TP53*. The somatic mutation profile of the tumors indicated that that loss of heterozygosity (LOH) at the *TP53* gene might have occurred during the differentiation of the founder clone into NEC, while a *SMAD4* mutation might have contributed to SCC development, indicating branching and subclonal evolution from common founder clone to both NEC and SCC. The mutation assessments provided valuable information to better understand the clonal evolution of metachronous cancers.

## Introduction

Esophagogastric junction (EGJ) carcinoma has attracted considerable attention because of the marked global increase in its incidence. The most common histology type, at this anatomic location, is adenocarcinoma. Adenocarcinoma of the EGJ is the sixth cause of cancer-related mortality, and the rate of morbidity is growing rapidly in both Western and Eastern countries (Parkin et al., 2001; Blaser and Saito, 2002; Pohl and Welch, 2005; Kusano et al., 2008). Neuroendocrine carcinomas (NECs) of the esophagus and stomach are rare; ranging from 0.01 to 0.08 cases per 100,000 people, each year (Savva et al., 2018). Generally, NEC in the gastrointestinal tract shows an inferior prognosis, and the median overall survival (OS), on average, is 1 year (Sorbye et al., 2013; Yamaguchi et al., 2014). Since the postoperative relapse rate is high even with complete resection, most patients, particularly those at an advanced stage, receive chemotherapy with cisplatin (CDDP) plus etoposide (VP-16) or irinotecan as the first-line therapy (Yamaguchi et al., 2014; Yoshida et al., 2019; Morita et al., 2020). It is generally considered that MIB-1 of ≥55%, in NEC lesions, shows worse prognosis than that when the MIB-1 is <55% (Sorbye et al., 2013).

Squamous cell carcinoma (SCC) of EGJ is a rare global occurrence, despite the frequency of esophageal SCC observed in the Japanese population. Neoadjuvant chemotherapy or chemoradiation therapy with cisplatin plus 5-fluorouracil (5-FU) is recommended as a first-line therapy for esophageal SCC (Ando et al., 2012; Noordman et al., 2018).

Whole-genome and whole-exome sequencing reveal the mutation profiles of both, esophageal and gastric cancers (Hu et al., 2016). As the tumors located at this site are histologically diverse, the mutational landscape of EGJ malignancies is yet to be elucidated in not only NECs and SCCs but also adenocarcinomas.

Herein, we present a case of SCC in a patient who was successfully treated and achieved a complete clinical response to treatment for NEC that arose in the EGJ. Moreover, we report the molecular profiles of the tumors, which suggest a common pathophysiological mechanism in both the tumors.

## Case Description

**Figure 1:**
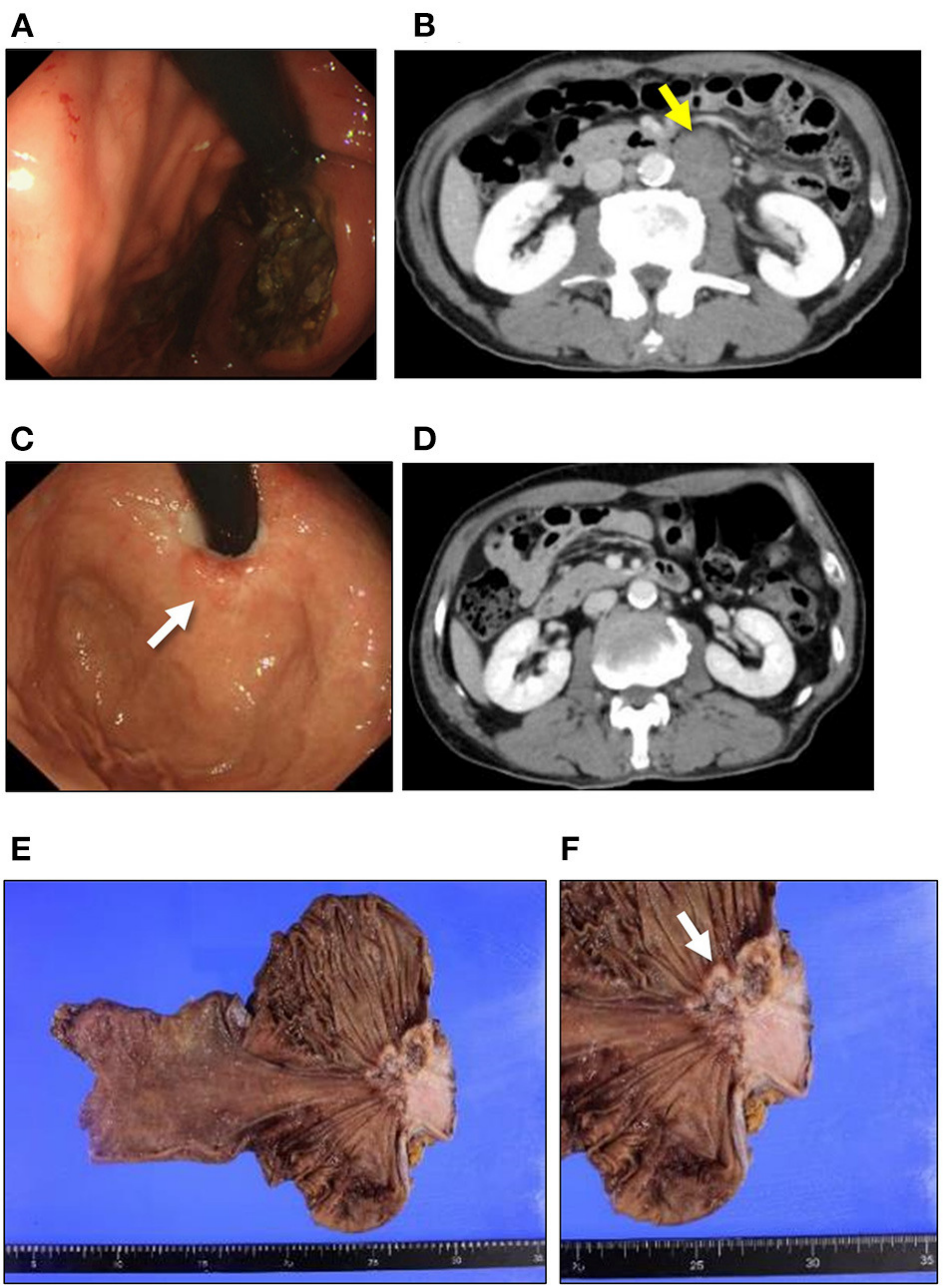
Clinical presentation of NEC and SCC. Esophagogastroduodenoscopy and CT findings in NEC **(A,B)**. **(A)** A large ulcerated mass was found in the esophagogastric junction. **(B)** CT imaging reveals para-aortic lymph node swelling (yellow arrow). Esophagogastroduodenoscopy and CT findings in SCC **(C,D)**. **(C)** A tiny, red-colored mucosal area in the center of the cicatrized lesion (white arrow). **(D)** CT imaging shows no distant metastasis after the chemotherapy for NEC. **(E)** Macroscopic findings for the resected specimen. No other regional/distant metastasis can be seen in the resected specimen. **(F)** An ulcerated mass at the esophagogastric junction can be observed in the resected specimen (white arrow). NEC, neuroendocrine carcinoma; SCC, squamous cell carcinoma; CT, computed tomography.

**Figure 2:**
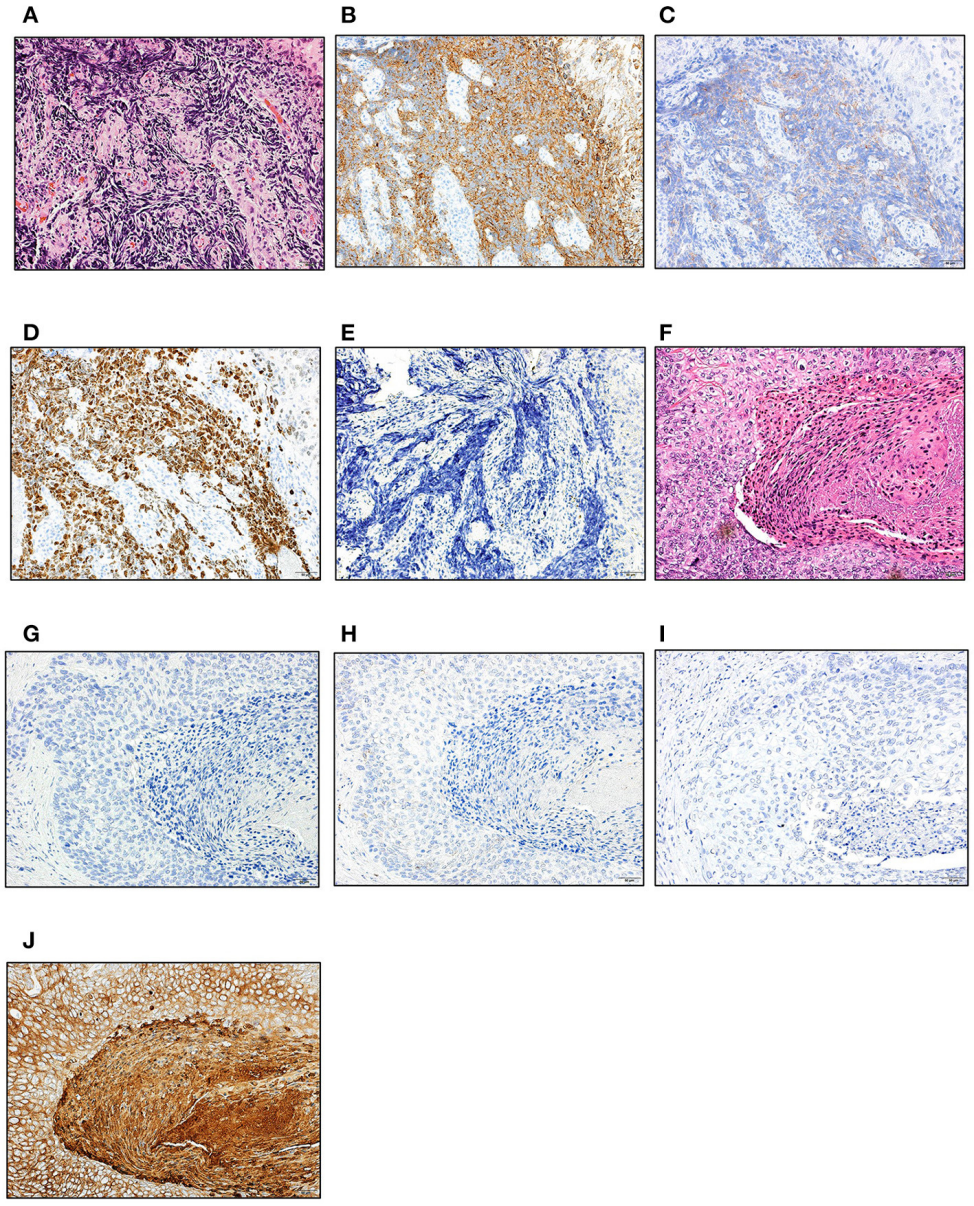
Histological evaluation of NEC and SCC. Hematoxylin and eosin, and immunohistochemical staining of the biopsy (NEC; **A–E**) and surgically resected specimen (SCC; **F–I**). **(A,F)** H-E staining; **(B,G)** synaptophysin; **(C,H)** CD56; **(D)** MIB-1; **(E,I)** p53; **(J)** CK5/6. Scale bars; 50 μm. NEC, neuroendocrine carcinoma; SCC, squamous cell carcinoma; H-E, hematoxylin and eosin.

A 67-year-old man was hospitalized as he presented with epigastric discomfort after experiencing symptoms for a few weeks. Physical examination revealed a non-distended abdomen, without any epigastric tenderness. He did not have a history of malignancy; however, he did have a family history of gastric cancer, and both his father and mother were afflicted. Blood chemistry tests showed an elevated lactate dehydrogenase level of 372 [U/L] (reference range: 115–245 [U/L]). Tumor markers, including carbohydrate antigen 19-9, neuron-specific antigen, cytokeratin 19 fragment, and pro-gastrin-releasing peptide, were within normal ranges. Esophagogastroduodenoscopy (EGD) revealed a large, ulcerated mass, 7 cm in diameter (Figure 1A). Subsequently, contrast-enhanced computed tomography (CT) imaging showed enlarged para-aortic lymph nodes, which was suggestive of lymph node metastasis from the primary lesion (Figure 1B). Biopsy of the primary tumor demonstrated round-shaped neoplastic cells with a high nucleus/cytoplasm ratio, consistent with pleomorphic, poorly differentiated carcinoma (Figure 2A). Immunohistochemistry (IHC) analysis revealed that cancer cells were positive for synaptophysin and negative for CD56, chromogranin A, and p53 (Figures 2B–E). The MIB-1 index was 80% (400 cells per 500 cells counted), and the mitotic rate was over 20 nuclei per 50 high power field. Based on the IHC and morphological characteristics, pathological assessments revealed a small-cell type NEC (Nagtegaal et al., 2020). Clinical staging was IV (T3, N1, M1), according to both the Union for International Cancer Control (UICC) (Brierley et al., 2017) and the European Neuroendocrine Tumor Society (ENETS) TNM staging (T3, N1, M1) systems for foregut (neuro)endocrine tumors (Rindi et al., 2006).

Since the patient had extra-regional lymph node metastases, cisplatin (CDDP) plus etoposide (VP-16) chemotherapy was administrated. After 10 courses of chemotherapy, the primary lesion and extra-regional lymph node metastasis were cicatrized after achieving a complete response, according to the National Cancer Institute Common Terminology Criteria for Adverse Events (CTCAE), Version 5.0. The regimen was well-tolerated by the patient for 39 weeks; however, the therapy was discontinued for the patient due to toxicity related to peripheral neuropathy. Thereafter, we switched the regimen to S-1 (tegafur/gimeracil/oteracil potassium) monotherapy. Three months after starting S-1 therapy, a follow-up EGD revealed a focal area of tiny, red-colored mucosal lesions at the center of the cicatrized lesion (Figure 1C), with no evidence of lymph node metastasis (Figure 1D). Re-biopsy of the tumor revealed keratinizing SCC, with no NEC components (Figure 2F). IHC was positive for Cytokeratin 5/6 (CK5/6) and negative for synaptophysin (Figures 2G–J). Additionally, ~60% of the entire SCC field was negative for SMAD4 staining (Supplementary Figure 1). We recommended the patient a treatment regimen involving total gastrectomy and lower esophagectomy, but the patient preferred chemoradiotherapy over surgical intervention. Second-line chemoradiotherapy with six cycles of 5-fluorouracil (5-FU) plus cisplatin in combination with intensity-modulated radiotherapy (50.4 Gy/28 Fr) was administered. After the therapy, the EGJ tumor persisted and showed slow longitudinal growth. The patient finally opted for total gastrectomy with lymph node dissection (Figures 1E,F). Colonoscopy was performed before the operation to exclude the possibility of multiple primary cancers, and no malignant or pre-malignant lesion, including polyposis along with gastrointestinal tract suggestive of hereditary cancer syndromes, was observed.

**Figure 3:**
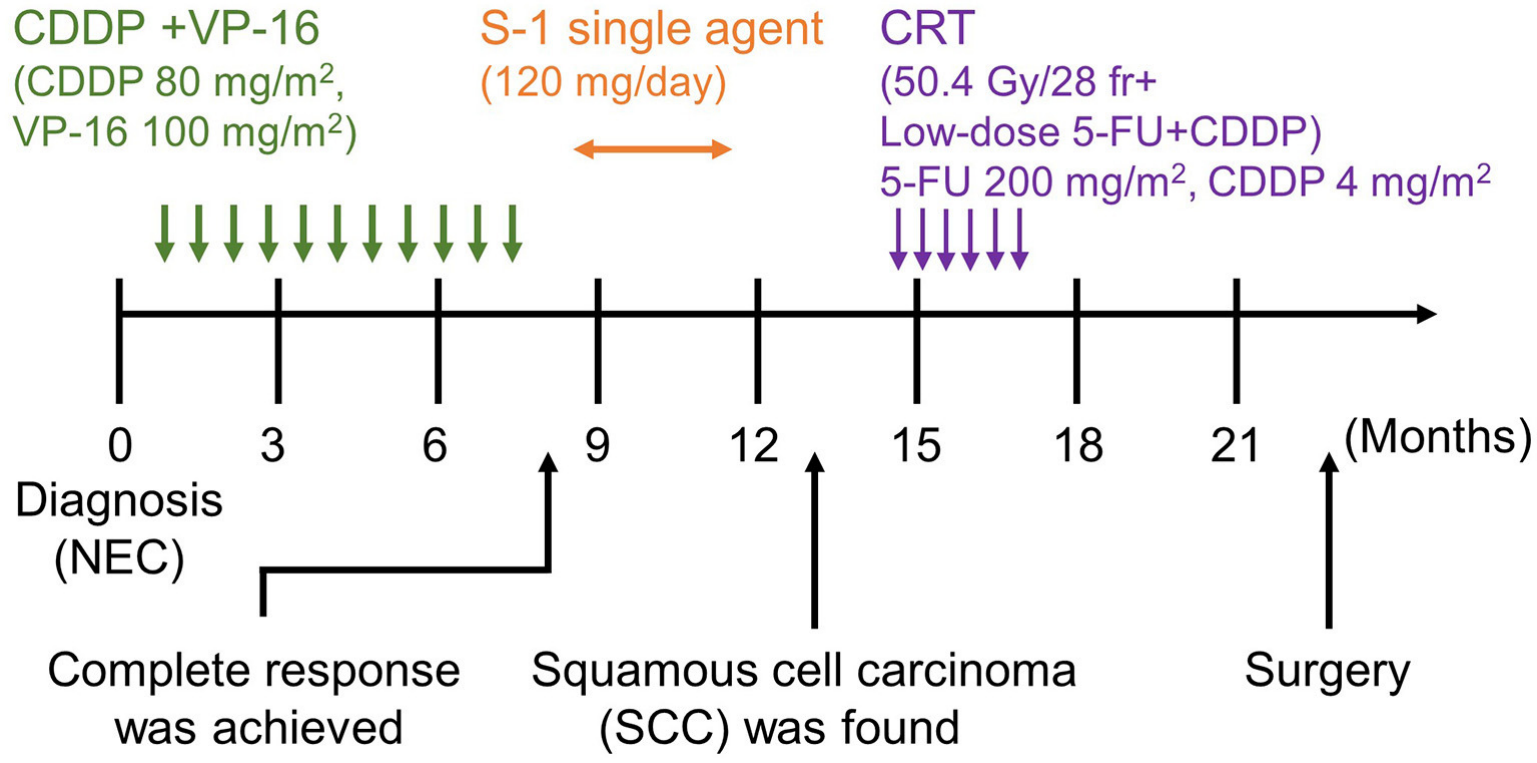
Timeline of the patient's clinical course. Following the diagnosis of NEC, CDDP+VP-16, and S-1 single-agent therapy was administered. CRT and surgical resection were performed after detecting SCC. NEC, neuroendocrine carcinoma; SCC, squamous cell carcinoma, CDDP+VP-16, cisplatin+etoposide therapy; S-1, tegafur/gimeracil/oteracil potassium; CRT, chemoradiotherapy.

The pathological examination of the resected specimen demonstrated SCC alone in the entire tumor. IHC analysis was negative for chromogranin A and synaptophysin, and positive for CK5/6. Additionally, CD56 staining was partially positive. Pathological staging was IIA (T3, N0, M0), and the patient survived without recurrence for >3 years after the initial presentation (Figure 3).

Given that the synchronous tumors were observed in close proximity, we hypothesized that both tumors may have shared a common neoplastic origin. To assess the genetic events that occurred during the clinical course and the histologic changes in the primary lesion, we characterized the primary tumor by targeted sequencing of formalin-fixed paraffin-embedded (FFPE) specimens. The samples for the DNA extraction included biopsy specimens from the primary NEC, the surgically resected SCC, and normal mucosa. Both tumors possessed identical *TP53* mutations at codon 192, identified as a pathogenic variant (p.Q192X; ClinVar accession, VCV000392993.2). Of note, the variant allele frequency of the *TP53* mutation in the NEC was 85.4%, whereas the frequency was 48.4% in the SCC component. Besides such allelic imbalance, only tumor cells from SCC harbored the *SMAD4* mutation (p.S517T). The *SMAD4* mutation was also not detected in the normal tissue. The *TP53* and the *SMAD4* mutations were not detected in the normal tissue. Two *STK11* variants (p.P281L, ClinVar accession; VCV000142115.10; p.F354L, ClinVar accession; VCV000376711.3) were found in both normal and tumoral tissues, and they are categorized as Benign/Likely Benign (Supplementary Table 1).

### Ethics Statement

The protocol for genetic analysis was approved by the institutional review board of Sunagawa City Medical Center (IRB approval #2019-18). Written informed consent was obtained from the patient before the genetic study, and for publication.

### Histological Evaluation

All histological sections were carefully examined, diagnosed, and reviewed by three pathologists (N.K, H.I, and Y.O), who assessed the distribution of the tumor compartments and performed IHC tests. Pathological staging of the surgical specimen was also performed by the above mentioned, three pathologists.

IHC staining of chromogranin A, synaptophysin, CD56, MIB-1, CK5/6, and p53 was performed with the antibodies as follows: anti-chromogranin A (anti-rabbit polyclonal antibody, 1:4; Nichirei Biosciences Inc., Tokyo, Japan); synaptophysin (27G12, 1:3; Nichirei Biosciences Inc., Tokyo, Japan); CD56 (MRQ-42, 1:5; Nichirei Biosciences Inc., Tokyo, Japan); MIB-1 (Ki-67, 1:400; Agilent Technologies, Inc., Santa Clara, CA, USA); CK5/6 (D5/16 B4, 1:250, Agilent Technologies, Inc., Santa Clara, CA, USA); anti-p53 (DO-7, 1:100; Cell Signaling Technology, Danvers, MA, USA); SMAD4 (B-8, 1:100; Santa Cruz Biotechnology, Santa Cruz, CA, USA).

### Radiological Evaluation

Radiological examinations were performed by a radiologist (N.T). All radiological data were assessed by him during the entire clinical course. The effects of treatment, during chemotherapy, was assessed according to the Response Evaluation Criteria in Solid Tumors (RECIST), version 1.1 (Eisenhauer et al., 2009).

### Samples and DNA Extraction

Genomic DNA was extracted from formalin-fixed, paraffin-embedded tissue specimens. Genomic DNA isolation and purification was obtained using the GeneRead DNA FFPE Kit (Qiagen, Hilden, Germany). The tissues used for analysis for NEC and SCC were from biopsy and resected specimens, respectively; the non-tumor tissue was retrieved from the non-cancer part of the resected specimen. The quantity of isolated genomic DNA was assessed using the Qubit 4.0 Fluorometer (Thermo Fisher Scientific, Waltham, MA, USA).

### Targeted Amplicon Sequencing

The mutation profiles of NEC and SCC were determined by targeted sequencing based on PCR amplicons, as described previously (Naito et al., 2018; Omori et al., 2019). In brief, 10 to 60 ng of genomic DNA was amplified using PCR, using Ion AmpliSeq Cancer Hotspot Panel v2 (Thermo Fisher Scientific, Waltham, MA, USA). Ion Ampliseq Cancer Hotspot Panel v2 is designed to amplify 207 amplicons covering major hotspots of 50 oncogenes, containing 440 DNA oligonucleotide primers designed for 220 amplicons (Thermo Fisher Scientific, Waltham, MA, USA; https://www.thermofisher.com/order/catalog/product/4475346#/4475346).

Sequencing was performed using an Ion S5 system and the Ion S5 Sequencing Kit (both were obtained from Thermo Fisher Scientific) according to the manufacturer's instructions. Sequence reads were demultiplexed, quality-filtered, and aligned to the human reference genome (Genome Reference Consortium Human Build 37) using Torrent Suite software (version 5.0.4; Thermo Fisher Scientific). Variants were identified using Variant Caller software (version 5.0.4.0; Thermo Fisher Scientific), and alignments were visually checked with Integrative Genomics Viewer software (version 2.3.59; Broad Institute, MA, USA).

## Discussion

Extrapulmonary NEC is rare, with more than 60% of such tumors developing along the gastrointestinal tract (Yao et al., 2008). Although, there are a few reports of mixed neuroendocrine non-neuroendocrine neoplasms (MiNEN) and NEC collision with SCC in the esophagus (Mendoza-Moreno et al., 2018; Choe et al., 2020); to the best of our knowledge, the current case is the first to be reported wherein a metachronous development of NEC and SCC is seen in the EGJ. In previous reports of MiNEN and collision carcinoma (Elkbuli et al., 2019; Kaneko et al., 2019; Choe et al., 2020), SCC was found on the surface of the mucosa, while NEC was found at a deeper level in the pathological sample. In the present case, NEC was found on the surface of the mucosa without any SCC components or adenocarcinoma, suggesting that NEC primarily occurred in the EGJ.

Since NEC frequently recurs after a complete resection (Katada et al., 2019), chemotherapy with cisplatin plus etoposide or irinotecan is commonly selected as the first-line therapy (Sorbye et al., 2013). Only 2% achieve a complete response with the cisplatin plus etoposide chemotherapy (Sorbye et al., 2013). Median OS varies depending on the site of the tumor; and the estimated OS with NEC in the esophagus and stomach are only 13.4–14.0 months and 11.0–13.3 months, respectively (Yamaguchi et al., 2014). In the present case, we achieved a complete response after administering 10 cycles of chemotherapy for NEC. Fortunately, the relative dose intensity was 100%, even though mild peripheral neuropathy developed after 6–7 cycles during the course. However, the prognosis of SCC in the EGJ and the esophagus has been reported to be 74.7 months (OS) and 29.9 months (recurrent free survival) (Shapiro et al., 2015). Neoadjuvant chemotherapy is the standard treatment option for esophageal SCC, and therefore, we decided to introduce 5-fluorouracil (5-FU) plus cisplatin in combination with intensity-modulated radiotherapy prior to the surgery.

IHC analysis of the tumor in the current case demonstrated a complete absence of p53 staining in both NEC and SCC compartments. Complete absence of p53 expression has been observed among samples where a loss-of-function mutation has occurred (Holstege et al., 2009). A detailed review of cases reported in a previous study indicated that *TP53* non-sense mutations, occurring before amino acid 213, resulted in its absence during IHC staining, supporting the lack of staining observed in our case (p.Q192X) (Kobel et al., 2016). In regards to the development of SCC, mutation analysis of the esophageal SCC demonstrated that a *TP53* non-sense mutation was found in ~20% of the esophageal SCC, supporting the notion that *TP53* is crucial for the development of esophageal SCC (Okuda et al., 2001; Gao et al., 2014). In contrast, the significance of the *SMAD4* mutation is yet to be determined in SCC, and its mutation frequency is lower than that in esophageal adenocarcinoma (Salem et al., 2018). Although the *SMAD4* S517T mutation has been categorized as VUS thus far, the variant may potentially be deleterious since immunohistochemical staining indicated reduced expression in part of the tumor.

**Figure 4:**
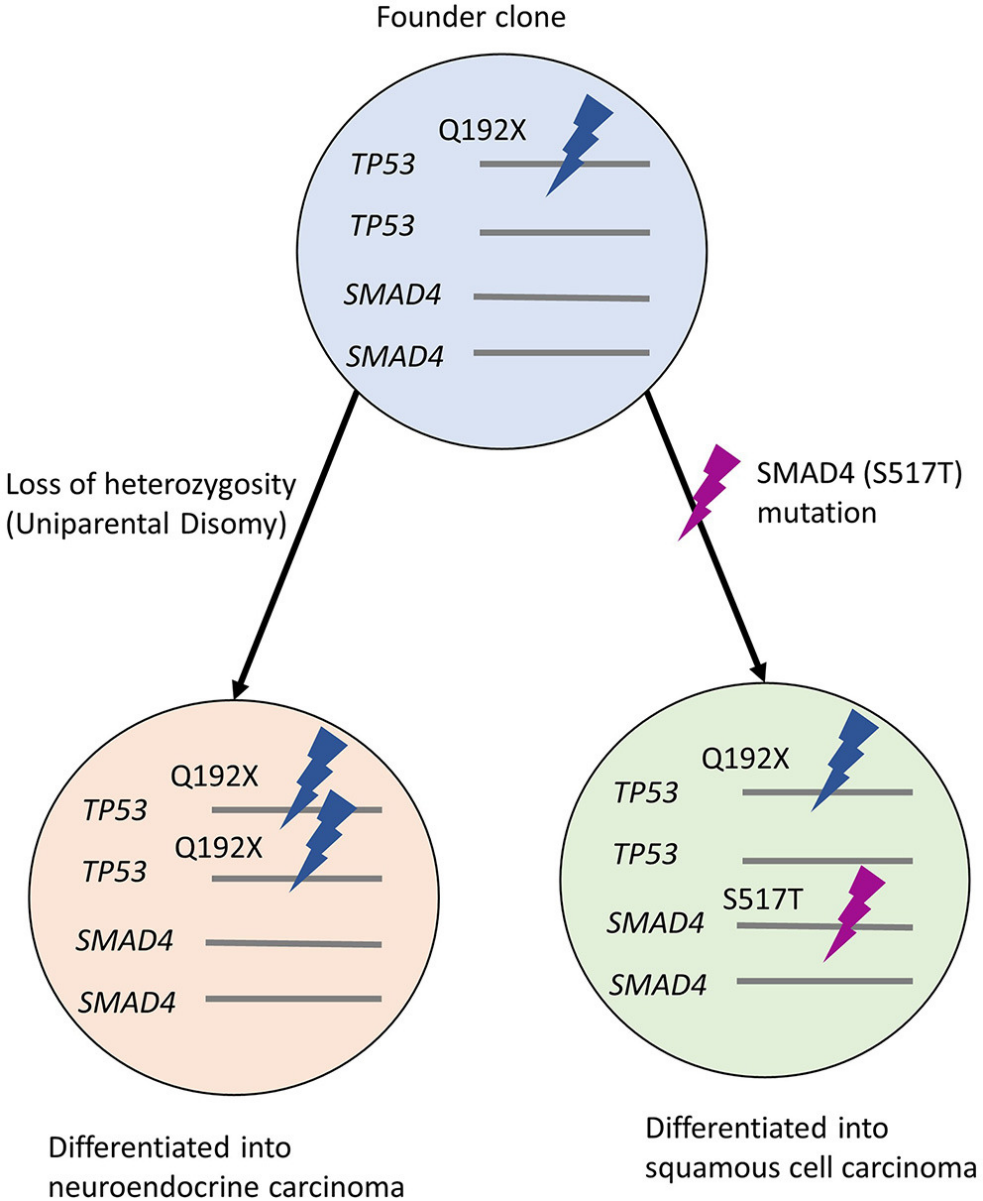
Hypothetical clonal evolution in this case. Founder clone developed uniparental disomy of *TP53* during proliferation, showing NEC morphology. The other population with *SMAD4* mutation shows the features of SCC. NEC, neuroendocrine carcinoma; SCC, squamous cell carcinoma.

Finally, we found that the variant allele frequency of *TP53* in NEC was twice as much as that in SCC. Considering that NEC had developed earlier than SCC, we speculated that a clonal population of cells with a *TP53* mutation had the ability to develop into both; NEC and SCC. During clonal expansion, the predominant subclone may have acquired *TP53* LOH, resulting in the emergence of poorly differentiated NEC. However, the latent branching population from the founder clone without biallelic inactivation of p53 may later acquire a *SMAD4* mutation, resulting in SCC features following the first-line chemotherapy (Figure 4). Recent comprehensive review and case reports showed the subset of non-small lung cell carcinomas with mutated *EGFR* returned as small cell lung carcinoma (Oser et al., 2015; Ren et al., 2020). Additionally, SCC could be transformed into the NEC along with the chemotherapy (Morita et al., 2016). However, the clonal evolution from NEC to SCC is extremely rare. Given the rarity, no decisive evidence can explain the mechanism of such morphological alterations. Chromosomal instability has been reported to play a vital role during the initiation of founder cancer cells (Spechler, 2013; Ye et al., 2020), and inflammatory processes such as Barrett's esophagus may be responsible for it. However, in this case, the endoscopic assessment did not support the presence of such chronic inflammations.

This case report has several limitations. First, total resection of the NEC was not performed; therefore, it is unknown whether intratumor heterogeneity, including the SCC component at a deeper level of the tumor, may have existed at the time of the diagnosis. Genotyping of the plasma circulating tumor DNA might have provided an opportunity to probe into this heterogeneity, during the evolution of cancer in the present case. Second, limited genetic profiling also limited our ability to infer clonal origins. The genes responsible for the hereditary cancer syndromes such as *BRCA1* and *BRCA2* are not included in the targeted sequencing. However, according to the familial history and the patients' history of cancer and pre-neoplastic diseases, it is unlikely that the NEC and SCC in EGJ had been developed as a result of pathogenic germline gene variants. Third, the precise assessment of copy number alterations and epigenetic analysis were not examined in this case.

## Conclusion

We described the first case of metachronous NEC and SCC that arose from the EGJ that was successfully treated with chemoradiation and surgical treatment. The sequential biopsy and surgical resection played a pivotal role in determining the histopathological features, which were needed to make appropriate changes in treatment. The mutation analysis of SCC showed *TP53* and *SMAD4* mutations, whereas that of NEC showed only *TP53* mutation. The unique clinical, pathological, and genetic tracing of the tumor during chemotherapy may have contributed to a better understanding of the genomic evolution of both NEC and SCC.

## Patient Perspective

Multidisciplinary treatment, including chemotherapy, chemoradiotherapy, and surgery can prolong the patient's survival with sequentially developed NEC following SCC in EGJ. The patient is still alive without any symptoms of recurrence for more than 3 years.

To the best of our knowledge, this is the first reported case wherein asynchronous SCC developed after the successful treatment of NEC. NEC in EGJ is extremely rare and is considered to be one of the worst prognoses among all gastrointestinal malignancies. Hence, the clinical course of our patient and the chosen treatment strategy is crucial for cases of NEC and SCC in EGJ, that may occur in the future.

In addition, the genetic analysis highlighted genetic evolution of NEC and SCC and suggests a common founder clone between these two tumors. It is clinically reasonable that the SCC was developed in the center scar of NEC.

## Data Availability Statement

The datasets for this article are not publicly available due to concerns regarding participant/patient anonymity. Requests to access the datasets should be directed to the corresponding author.

## Ethics Statement

The studies involving human participants were reviewed and approved by the Sunagawa City Medical Center. The patients/participants provided their written informed consent to participate in this study. Written informed consent was obtained from the individual(s) for the publication of any potentially identifiable images or data included in this article.

## Author Contributions

HS, MK, YY, KA, and YMi: design of the study. HS, KA, YOn, and YMi: drafting of the manuscript. NK, HI, YOm, and AL: histological analysis. NT: radiological imaging evaluation. HS, MG, YMa, KA, MF, YOn, MG, and YMi: tumor sequencing and data interpretation. TS, MK, HH, HS, MH, KT, MY, YY, MF, TH, and TO: coordination of clinical care and specimen procurement and analysis. HS, YY, and YMi: design of the study. All authors have read and approved the manuscript.

## Funding

This work was supported by the Japan Society for the Promotion of Science (JSPS) KAKENHI (grant number 17K09472 to YMi, 19K17480 to HS) and by the Suhara Memorial Foundation (to YMi). The role of the funding body was to provide a budget for genetic analysis.

## Conflict of Interest

YMi and YOn have received funding from Hitachi High-Tech Inc. The remaining authors declare that the research was conducted in the absence of any commercial or financial relationships that could be construed as a potential conflict of interest.

## Publisher's Note

All claims expressed in this article are solely those of the authors and do not necessarily represent those of their affiliated organizations, or those of the publisher, the editors and the reviewers. Any product that may be evaluated in this article, or claim that may be made by its manufacturer, is not guaranteed or endorsed by the publisher.

## Supplementary Material

Supplementary Figure 1 SMAD4 immunohistochemical staining on SCC. Hematoxylin and eosin, and immunohistochemical staining of the SCC. **(A)** H-E staining; **(B)** SMAD4 staining. Scale bars; 100 μm.

Supplementary Table 1 Mutation profiles of the primary and recurrent EGJ lesions.

## Abbreviations

NEC: Neuroendocrine carcinoma
EGJ: Esophagogastric junction
SCC: Squamous cell carcinoma
IHC: Immunohistochemistry
LOH: Loss of heterozygosity
5-FU: 5-fluorouracil
CT: Computed tomography
CK5/6: Cytokeratin 5/6
CDDP: Cisplatin
VP-16: Etoposide
NEN: Neuroendocrine neoplasms
RECIST: Response Evaluation Criteria in Solid Tumors
CTCAE: National Cancer Institute Common Terminology Criteria for Adverse Events
PCR: Polymerase chain reaction
MiNEN: Mixed neuroendocrine-non-neuroendocrine neoplasms.
