# Supplementary material for: Case Report: A Rare Case of Esophagogastric Junctional Squamous Cell Carcinoma After the Successful Treatment of Neuroendocrine Carcinoma: Clonal Tumor Evolution Revealed by Genetic Analysis
Source: Front Genet. 2021 Sep 15;12:608324. doi: 10.3389/fgene.2021.608324 (PMC8489402; doi:10.3389/fgene.2021.608324)
Supplement: Supplementary file 1 [file Presentation_1.PDF]

CARE case report guidelines

CARE Checklist of information to include when writing a case report

(cc) BY-NC-ND

Topic	Item	Checklist item description	Reported on Line
Title	1	The diagnosis or intervention of primary focus followed by the words "case report"	1-3
Key Words	2	2 to 5 key words that identify diagnoses or interventions in this case report, including "case report"	52-53
Abstract (no references)	3a	Introduction: What is unique about this case and what does it add to the scientific literature?	28-29
	3b	Main symptoms and/or important clinical findings	29-30
	3c	The main diagnoses, therapeutic interventions, and outcomes	31-40
	3d	Conclusion—What is the main "take-away" lesson(s) from this case?	48-49
Introduction	4	One or two paragraphs summarizing why this case is unique (**may include references**)	67-74
Patient Information	5a	De-identified patient specific information.	80-81
	5b	Primary concerns and symptoms of the patient.	81-82
	5c	Medical, family, and psycho-social history including relevant genetic information	82-83
	5d	Relevant past interventions with outcomes	82
Clinical Findings	6	Describe significant physical examination (PE) and important clinical findings.	81-89
Timeline	7	Historical and current information from this episode of care organized as a timeline	81-119
Diagnostic Assessment	8a	Diagnostic testing (such as PE, laboratory testing, imaging, surveys).	83-89
	8b	Diagnostic challenges (such as access to testing, financial, or cultural)	89-111
	8c	Diagnosis (including other diagnoses considered)	89-98, 115-118
	8d	Prognosis (such as staging in oncology) where applicable	118-119
Therapeutic Intervention	9a	Types of therapeutic intervention (such as pharmacologic, surgical, preventive, self-care)	99-115
	9b	Administration of therapeutic intervention (such as dosage, strength, duration)	99-115
	9c	Changes in therapeutic intervention (with rationale)	103-115
Follow-up and Outcomes	10a	Clinician and patient-assessed outcomes (if available)	117-119
	10b	Important follow-up diagnostic and other test results	120-129
	10c	Intervention adherence and tolerability (How was this assessed?)	103-115
	10d	Adverse and unanticipated events	103-115
Discussion	11a	A scientific discussion of the strengths AND limitations associated with this case report	194-216
	11b	Discussion of the relevant medical literature **with references**.	174-203
	11c	The scientific rationale for any conclusions (including assessment of possible causes)	194-210
	11d	The primary "take-away" lessons of this case report (without references) in a one paragraph conclusion	204-210
Patient Perspective	12	The patient should share their perspective in one to two paragraphs on the treatment(s) they received	227-237
Informed Consent	13	Did the patient give informed consent? Please provide if requested	Yes ☑ No ☐
