# Supplementary figures and images for: Case Report: A Rare Case of Esophagogastric Junctional Squamous Cell Carcinoma After the Successful Treatment of Neuroendocrine Carcinoma: Clonal Tumor Evolution Revealed by Genetic Analysis
Source: Front Genet. 2021 Sep 15;12:608324. doi: 10.3389/fgene.2021.608324 (PMC8489402; doi:10.3389/fgene.2021.608324)

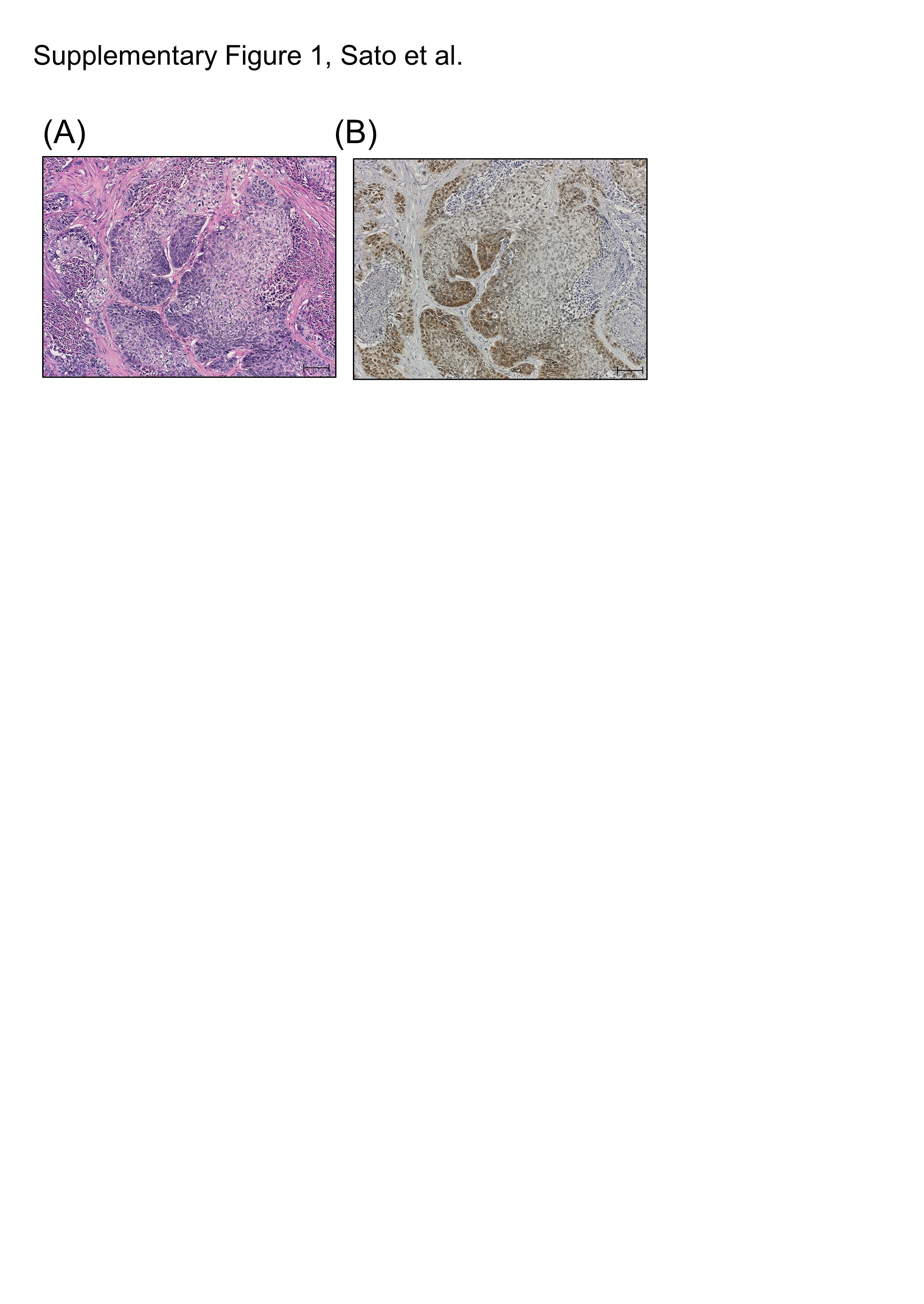

Supplement: Supplementary Figure 1 — SMAD4 immunohistochemical staining on SCC. Hematoxylin and eosin, and immunohistochemical staining of the SCC. (A) H-E staining; (B) SMAD4 staining. Scale bars; 100 μm. [file Image_1.JPEG]
